# Differentially expressed genes in adipocytokine signaling pathway of adipose tissue in pregnancy

**DOI:** 10.4236/jdm.2013.32013

**Published:** 2013-05

**Authors:** Dotun Ogunyemi, Jun Xu, Arnold M. Mahesan, Steve Rad, Eric Kim, Jacqueline Yano, Carolyn Alexander, Jerome I. Rotter, Y.-D. Ida Chen

**Affiliations:** 1 Department of Obstetrics and Gynecology, Cedars-Sinai Medical Center, Los Angeles, USA; 2 Department of Medical Education, David Geffen School of Medicine at UCLA, Los Angeles, USA; 3 Department of Medical Genetics, Cedars-Sinai Medical Center, Los Angeles, USA; 4 Department of Endocrinology, Cedars-Sinai Medical Center, Los Angeles, USA

**Keywords:** Adipose Tissue, Pregnancy, Adipocytokine Pathway, Insulin Resistance

## Abstract

**Objective:**

To profile the differential gene expression of the KEGG Adipocytokine Signaling pathway in omental compared to subcutaneous tissue in normal pregnancy.

**Study Design:**

Subjects included 14 nonobese, normal glucose tolerant, healthy pregnant women. Matched omental and subcutaneous tissue were obtained at elective cesarean delivery. Gene expression was evaluated using microarray and validated by RT-PCR. Differential gene expression was defined as ≥1.5 fold increase at p < 0.05.

**Results:**

Six genes were significantly downregulated with two upregulated genes in omental tissue. Downregulation of Adiponectin and Insulin Receptor substrate, key genes mediating insulin sensitivity, were observed with borderline upregulation of GLUT-1. There were downregulations of CD36 and acyl-CoA Synthetase Long-chain Family Member 1which are genes involved in fatty acid uptake and activation. There was a novel expression of Carnitine palmitoyltransferase 1C.

**Conclusion:**

Differential gene expression of Adipocytokine Signaling Pathway in omental relative to subcutaneous adipose tissue in normal pregnancy suggests a pattern of insulin resistance, hyperlipidemia, and inflammation.

## 1. INTRODUCTION

Pregnancy is associated with changes in the regulation of glucose metabolism and the development of insulin resistance particularly during the second and third trimesters [1] of all pregnancies.

However, there is no consensus regarding the precise pathophysiology of insulin resistance, which is perceived as causative of gestational diabetes mellitus. It has also been suggested that the observed increase in body fat mass from the 1^st^ trimester throughout pregnancy may be causally associated with decrease in insulin sensitivity [2].

Intra-abdominal (omental or visceral) adipose has been implicated as a major factor in pathophysiology of insulin resistance [3]. It has been observed that visceral fat accumulation increases during pregnancy [4]. Further, Martin et al have noted a significant association between visceral adipose tissue-depth above upper quartile value on ultrasound with positive glucose challenge test in later pregnancy, with no such associations seen for subcutaneous adipose tissue [5].

**Figure 1:**
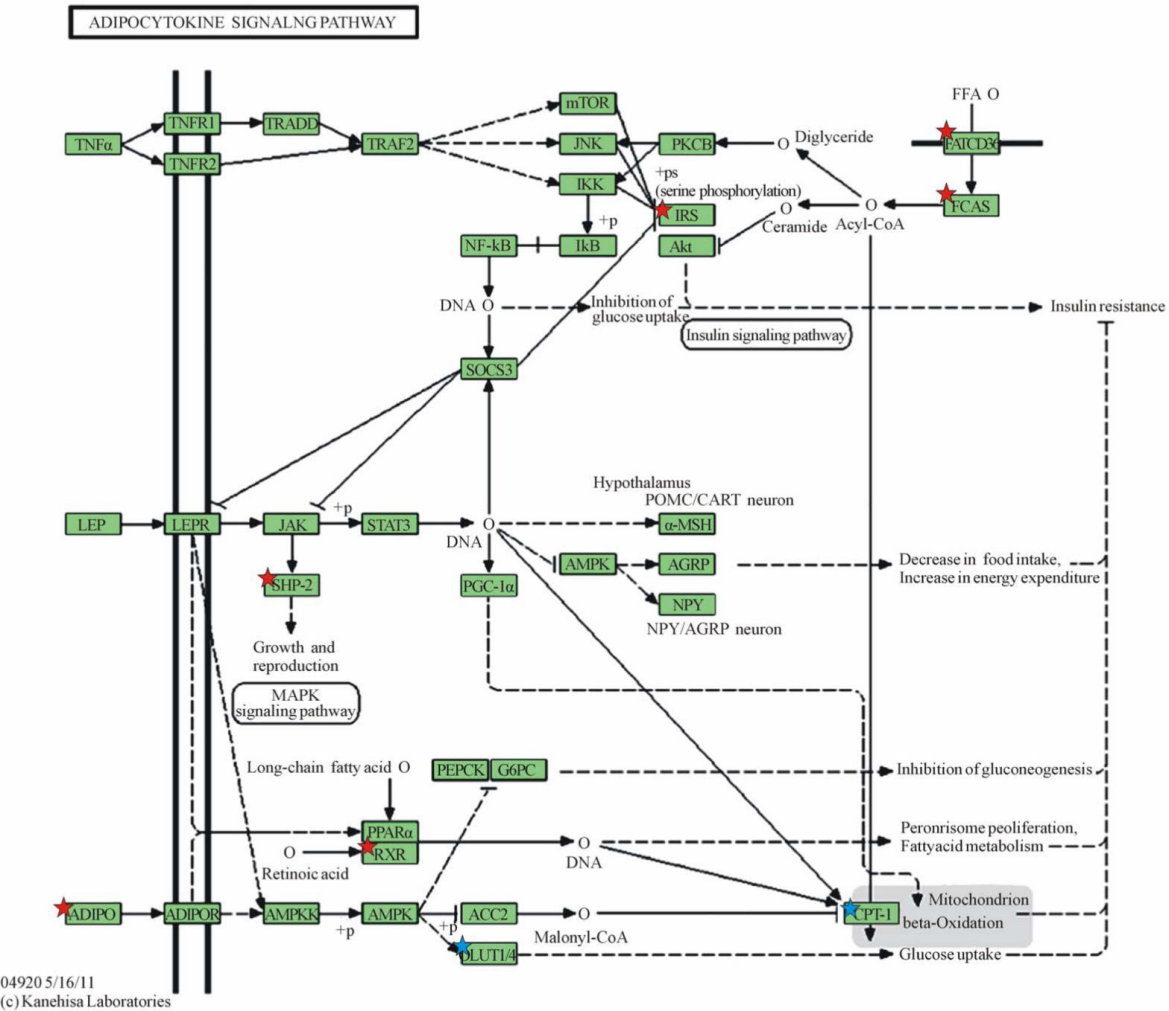
KEGG Adipocytokine Signaling Pathway. Downregulated genes are those marked with red stars: ADIPO (Adiponectin), SHP2 (Protein Tyrosine Phosphatase, Non-Receptor Type 11—PTPN11), RXR (Retinoid X-Receptor, alpha), IRS1 (Insulin Receptor Substrate 1), FATCD36 (CD36 molecule, thrombospondin receptor), FACS (acyl-CoA synthetase long-chain family member 1—ACSL1); Upregulated genes are marked with blue stars as they appear in the KEGG gene listing: SLC20A1 (shown as GLUT-1) and CPT1C (listed under CPT-1). Legend of KEGG gene symbols and full gene names for differentially expressed genes appears below [6,7]. Legend of differentially expressed genes in KEGG adipocytokine signaling pathway (Figure 1).

Adipose tissue is metabolically active, producing adipocytokines that exert effects in energy homeostasis and insulin resistance. The KEGG Adipocytokine Signaling Pathway (Figure 1) [6,7] describes signaling cascades arising from the adipocytokines that have been implicated in insulin resistance and sensitivity: TNF-alpha, leptin and adiponectin. Leptin is increased in pregnancies complicated by pre-eclampsia, BMI > 25, gestational diabetes, or hyperinsulinemia [8–11]. Adiponectin is an adipocytokine that has been demonstrated to have antiatherogenic, anti-inflammatory, and antidiabetic roles [12]. Adiponectin has been correlated with insulin sensitivity [13]. Decreased serum adiponectin is consistently seen in women with gestational diabetes independent of body mass index, strongly suggesting a role for adiponectin in modulating insulin resistance during gestation [14]. In late pregnancy, the placenta Produces and secretes adiponectin [15]. Adiponectin has also been observed to play a role in adapting energy metabolism at the maternal-fetal interface [15]. Metabolic changes associated with expanding adipose tissue are linked to subclinical inflammation which has been demonstrated to be mediated by cytokines such as TNF-alpha and IL-6 [16]. A signaling cascade arising from TNF-alpha is shown in the KEGG pathway (Figure 1), resulting in increased insulin resistance.

There is a paucity of studies examining differential gene expression of the adipocytokine signaling pathway. In the present investigation, we examine differential gene expression in omental and subcutaneous adipose depots in late-term gravid women as relevant to the adipocytokine signaling pathway. We performed this analysis to further elucidate the relative role of the various adipose depots in glucose homeostasis in normal pregnancy in non-obese women. We hypothesize that we will observe changes in differential gene expression in omental adipose consistent with a pattern of relative insulin resistance.

Identifying mechanisms of insulin resistance associated with various adipose tissue depots may help to better elucidate the pathogenesis of insulin resistance, which may have practical implications in the management of diabetes and its complications.

## 2. MATERIALS AND METHODS

### 2.1. Adipose Tissue Collection

This is a prospective study on investigating the pathophysiology of insulin resistance and diabetes in pregnancy. We recruited pregnant women between 18 to 45 years during prenatal care. Pregnant women were eligible for this study if they had a BMI < 30; were to be scheduled for an elective cesarean delivery and had a normal glucose challenge test performed during prenatal care. Informed consent was obtained, demographic information was obtained, and patients underwent routine prenatal care. During the cesarean delivery; maternal and cord blood, subcutaneous and omental adipose tissue samples were obtained. The adipose tissues were snap frozen in liquid nitrogen and stored in −80°C freezer. The patient recruitment and study protocol was approved by Cedars-Sinai Medical Center Institutional Review Board. In this report, we focus on the KEGG adipocytokine pathway. Other KEGG pathways that are relevant to insulin resistance and diabetes in pregnancy that we are studying for other reports are complement and coagulation; Cytokine-cytokine receptor interaction, Arachidonic acid metabolism and insulin signaling pathways.

### 2.2. RNA Extraction and Labeling

Total RNA was extracted from adipose tissues using RNeasy Lipid Tissue kit from Qiagen following the manufacturer’s instructions. Briefly, about 30 mg of frozen adipose tissues were resected and disrupted in 1 ml QIAzollysis reagent with handheld tissue homogenizer (VWR). The homogenates were incubated at room temperature for 5 min followed by mixing with 200 ul chloroform (Sigma Aldrich) and centrifuging at 12,000 g for 15 min at 4°C. The top aqueous phase of the homogenates containing total RNA were then mixed with 600 ul 70% ethanol (Sigma Aldrich) and loaded onto RNeasy Mini spin column to capture the RNA onto the column’s membrane after brief centrifugation at about 10,000 g. On column DNA digestion with RNase-free DNase I set (Qiagen) was carried out to get rid of carry-over genomic DNA. RNA was washed twice and eluted from the membrane with RNase-free water. The concentration and purity of RNA were measured with UV spectrometer (Bio-rad). RNA integrity was checked with Agilent Bio-analyzer with RNA 6000 nano kit.

IlluminaTotalPrep RNA Amplification kit (Ambion) was used to generate biotin-labeled cRNA from total RNA. Briefly, first strand cDNA was made from about 300 ng of total RNA with oligoT7 primer, dNTP, RNase inhibitor, and ArrayScript reverse transcriptase in first strand buffer, incubated at 42°C for 2 hours. Then second strand cDNA was synthesized at 16°C for 2 hours with DNA polymerase, dNTP mix, RNase H, and second strand buffer. After purification of cDNA with cDNA-Pure magnetic beads, *in vitro* transcription (IVT) was carried out with biotin-labeling NTPs and IVT enzymes to synthesize biotin-labeled cRNA at 37°C for 14 hours. Finally, cRNA was purified using RNA binding magnetic beads and eluted with cRNA elution buffer.

### 2.3. Microarray Hybridization Staining and Scanning

Biotin labeled cRNA (750 ng) was loaded on to Illumina HumanHT-12 V4.0 Expression BeadChip, which comprises probes targeting more than 47,000 transcripts across whole genome, in hybridization buffer with hybridization controls and incubated for 16 hours at 58°C. After hybridization, BeadChips were washed, blocked with blocking buffer and stained with streptavidin-Cy3 (Amersham Biosciences). Images of stained BeadChips were captured by IlluminaBeadArray Reader.

### 2.4. Microarray Image Processing and Data Analysis

Images of BeadChip were loaded into IlluminaGenomeStudio for quality determination by evaluation of the present call percentages, signal intensities of hybridization control, biotin controls, and negative controls. Quantile normalization was applied during data processing in GenomeStudio. Signal intensities of each gene/probe were exported into Partek Genomic Suite 6.5 for high level data analyses. Partek built-in ANOVA model was used to evaluate the impacts of various variables in the study such as different adipose tissues, patient ethnicity, patient-to-patient variability, etc., on the expression data set. Differentially expressed genes (DEGs) of omental fat versus subcutaneous fat were defined as 1.5-fold difference in either direction plus pair-wise *t*-test with Benjamini & Hochberg adjusted p < 0.05. Functional classification and pathway analysis were carried out using DAVID Bioinformatics Resources 6.7 (National Institute of Allergy and Infectious Diseases, NIAID, NIH) [13,14].

**Figure 2:**
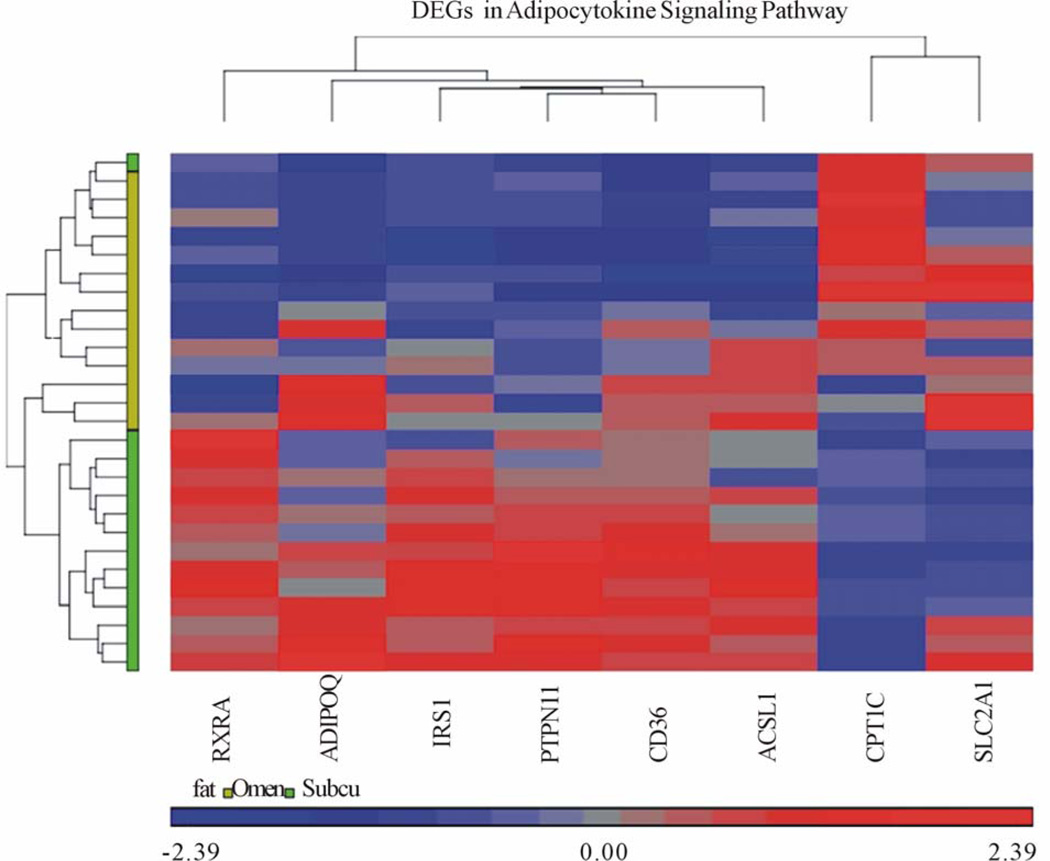
Heat map of differentially expressed genes (DEGs) in Adipocytokine Signaling Pathway. Each column on the horizontal axis represents a tissue sample. (14 subjects) × (2 tissue samples each, omental and subcutaneous) = 28 total tissue samples. Each row on the vertical axis represents one of the 8 DEGs.

A heat-map is a rectangular tiling of data with cluster trees appended to its margins [15]. Weinstein describes the heat map as follows: in the case of gene expression data, the color assigned to a point in the heat map grid indicates how much of a particular RNA or protein is expressed in a given sample. The gene expression level is generally indicated by red for high expression and either green or blue for low expression. Coherent patterns (patches) of color are generated by hierarchical clustering on both horizontal and vertical axes to bring like together with like. Cluster relationships are indicated by tree-like structures adjacent to the heat map, and the patches of color may indicate functional relationships among genes and samples [16]. In our heat-map (Figure 2), tissue samples are displayed on the x-axis while genes are listed on the y-axis. Higher gene expression levels are indicated by red and lower expression levels are indicated by blue or grey. On the vertical and horizontal margins of the tiling there are hierarchical cluster-trees arranging the rows and columns so that similar rows or columns of gene expression levels are near each other [15].

### 2.5. Real-Time PCR Validation of Microarray Results

We used quantitative RT-PCR to verify the differential expression level of several genes based on either their biological functions or chromosomal locations. Confirmation of genes not present in the adipocytokine pathway helped to control for stochastic biological sampling error exacerbated by multiple testing [12]. Procedurally, selected differentially expressed mRNAs were validated with real-time PCR. RNA was first reverse-transcribed to cDNA using iScript Reverse Transcription Supermix for RT-qPCR (Bio-rad). About 10 ng cDNA were mixed with gene specific primers (Table 1) and Platinum Sybr Green qPCRSuperMix-UDG (Invitrogen). Real-time PCR was carried out in ABI 7000 Sequence Detector (Applied-Biosystems) for 40 cycles. The expression level of human *GAPDH* gene was used as an internal control. Fold changes of omental adipose tissue group versus subcutaneous group were calculated using CT method.

## 3. RESULTS

The study is based on 14 patients with a body mass index (BMI) <30 who had an elective Cesarean section performed between 2008 and 2010. The mean age was 32.1 years (SD = 6.21), the mean BMI was 23.3 (SD = 2.88), the mean gestational age at delivery was 39 + 0/7 weeks (SD = 4.37 days), and the mean birth weight was 3240 grams (SD = 384.7 grams) (Table 2). Regarding the ethnic distribution: 40% were Hispanic, 27% African American, 13% Asian, and 20% were non-Hispanic whites.

**Figure 3:**
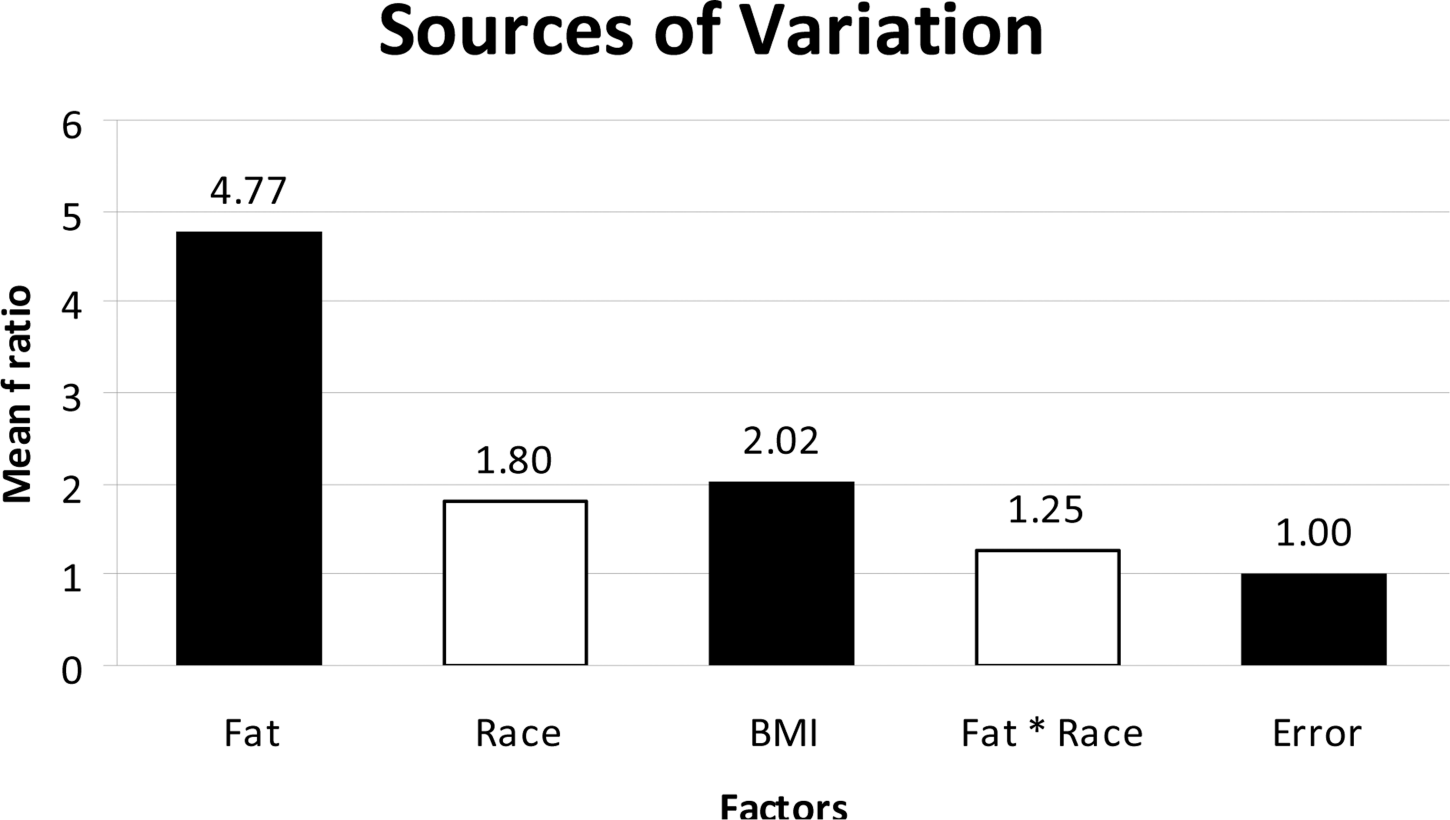
ANOVA model used to evaluate the impacts of the potential confounding variables: fat, race, BMI, fat and race in the differentially expressed genes in the study population. The horizontal column indicates the Mean F Ratio while the various factors analyzed in the multi factor ANOVA are listed horizontally along the x-axis. Any factor that has a Mean F Ratio more than error (1.00) is considered “significant”.

Categorical factors including fat, BMI, and race were analyzed using ANOVA to estimate the contribution of each factor to our data set and allow the identification of significant sources of variation (Figure 3). Any factor having a ratio greater than an error of 1 was considered significant. As seen in Figure 3, adipose tissue had almost a five-fold effect with an F ratio of 4.77. BMI had a ratio of 2.02 which highlights the impact of BMI and for this reason; we apriori planned to minimize the effect of obesity by excluding from the study any women with a BMI of 30 that would be classified as obesity. Race had a ratio of 1.8. Although the race factor has a value higher than can be contributed to error, when we compared the interaction of race with adipose tissue on differential gene expression using a mixed model ANOVA (column 4) the effect was only 1.25, which is borderline, and suggests minimal to no interference of race on the differential gene expression of adipose tissue.

Six genes of the KEGG Adipocytokine Signaling Pathway orthography were significantly downregulated in omental adipose relative to subcutaneous adipose tissue, (Table 3). Two genes were also found to be significantly upregulated (Table 3). The gene most downregulated in omental adipose tissue was CD36, by a factor of 2.05839. The significantly downregulated genes were found in four cascades within the Adipocytokine Signaling Pathway. In the pathway with initial signaling by Adiponectin; both Adiponectin and Retinoid × receptor alpha were significantly downregulated. In the cascade initiated by TNF-alpha; Insulin Receptor Substrate 1 (IRS1), was significantly downregulated with no significant differential expression of TNF-alpha noted. In the pathway with initial signaling by Leptin, Protein Tyrosine Phosphatase Non-Receptor Type 11 (PTPN11 also known as SHP2), was also significantly downregulated without significant differential expression of Leptin. Finally, both CD36 (FATCD36) and acyl-CoA Synthetase Long-chain Family Member 1 (ACSL1, seen in Figure 2 as FACS) involved in the cascade of free fatty acid uptake and activation were downregulated in omental versus subcutaneous tissue.

Solute Carrier Family 2, Member 1(SCLC2A1) also known as GLUT1 was upregulated with a borderline fold increase of 1.59. GLUT1 is a minor facilitated glucose transporter; however no differential expression of GLUT-4 which is the major glucose transporter was observed. Also upregulated was carnitine palmitoyltransferase 1C (CPT1C); this gene is included in the KEGG pathway as part of the CPT-1 gene complex, which comprises the 3 isoforms of CPT1A; CPT1B and CPT1C. The CPT-1 genes are generally involved in fatty acid oxidation but the specific function of CPT1C isoform is poorly understood.

Our heat-map shows significantly expressed genes and their relative expression in both subcutaneous and omental adipose tissue samples. The heat map analysis was able to differentiate omental and subcutaneous tissues via hierarchical clustering. The tissues clump together as per the color bar beneath the horizontal cluster tree, the grouping supports the hypothesis that the adipocytokine pathway is activated in the omental tissue (Figure 2).

Table 4 shows the quantitative RT-PCR results for the 7 positional candidate genes, which confirms the differential expression of all five upregulated genes and the two downregulated genes [19]. For example, for the IRS1 gene, the microarray fold change was −1.77, and the RT-PCR fold change was −1.48. We utilized genes not present in the adipocytokine pathways for external validation.

## 4. DISCUSSION

The objective of this study was to determine differentially expressed genes in omental when compared to subcutaneous adipose tissue in the KEGG Adipocytokine Signaling Pathway orthography, which may be associated with insulin resistance, inflammation, or adverse pregnancy outcomes.

Figure 1 depicts KEGG Adipocytokine Signaling Pathway and shows the cascades signaled by Adiponectin (ADIPO) and Leptin (LEP), the two major adipocytokines, and TNF-alpha (TNFα), a major inflammatory cytokine. Also depicted is a cascade leading to the uptake and activation of Free Fatty Acids via CD36 (FATCD36) and activation via acyl-CoA synthetase long chain family 1 (FACS).

The KEGG pathway cascade arising from TNF-alpha stimulation results in negative regulation of the IRS1 gene. TNF-alpha is an important inflammatory mediator associated with insulin resistance [20], and increased TNF-alpha has been linked to gestational diabetes [21, 22]. A critical function of the IRS1 is to interact with the Insulin Receptor, enabling the metabolic actions of insulin by signaling the PI3K pathway [23]. Thus, IRS1 is a pivotal gene in insulin sensitivity [24–26]. Of note is that IRS1 gene variations have been associated with increased in visceral to subcutaneous fat ratio as determined by computerized tomography of the abdomen [24]. In our study, IRS1 was downregulated in the omental adipose tissue, suggesting increased insulin resistance in that tissue. Another study showed significantly reduced IRS1 mRNA levels in adipocytes from obese compared to lean non-pregnant Pima Indians. Further, a role for IRS1 in the pathogenesis of type 2 diabetes has been suggested because of differential expression of IRS1 variants [23]. We did a PUBMED search using the following phrases “insulin receptor”, insulin receptor protein”, “adipose tissue”, “omentum”, “pregnancy”, “pregnancy complications”, “diabetes in pregnancy” and we could not find any report on downregulation of IRS1 in omental compared to subcutaneous tissue in non-obese healthy, nondiabetic pregnant women; consequently we believe that this finding is a contribution to the literature.

Protein Tyrosine Phosphatase, Non-Receptor Type 11 (PTPN11 also known as SHP2) was also downregulated in the omental adipose depot. The KEGG orthography notes SHP2 (PTPN11) is involved in signaling the MAPK signaling pathway, with consequences to cell growth and development. This is through activation by SHP2 of MAPK/ERK in response to leptin [29]. It has also been demonstrated that interactions between SHP2 and IRS1 promotes binding of IRS1 to the insulin receptor. SHP2, then, acts as an adaptor for insulin receptor and IRS1 forming a multiprotein signaling complex involving all three proteins to enhance glucose uptake. Downregulation of expression of SHP2, in the omental adipose depot [30] may serve to further predispose to insulin resistance.

In another signaling cascade, Adiponectin (ADIPO) was found to be downregulated in the omental depot in our study. As shown in the KEGG pathway, Adiponectin signals increased glucose uptake via the GLUT transporters [29,30]. To establish the involvement of adiponectin in insulin resistance in pregnancy, Cortelazzi *et al.* showed significantly lower adiponectin serum levels in women with GDM than in nondiabetic women at the same gestational ages [16]. Other studies have also shown decreases in serum adiponectin in late normal pregnancy [31,32]. Reports have shown that adiponectin is associated with improved glucose sensitivity, central body fat distribution, and that serum adiponectin concentrations are determined mainly by visceral adipose [33]. Our findings of differential downregulation of adiponectin in omental tissue in healthy non-obese pregnant women adds to this literature and support the association between visceral fat and increased insulin resistance.

Retinoid × Receptor, alpha (RXR) was also downregulated in omental adipose in our study. The KEGG Pathway shows that RXR forms a heterodimer with PPAR-alpha, and that PPAR-alpha is activated via leptin and adiponectin stimulation [34,35]. Activation of PPAR-alpha promotes uptake and utilization of fatty acids by upregulation of genes in fatty acid transport and beta-oxidation [36]. Downregulation of RXR can then lead to diminished fatty acid uptake and utilization in omental adipose tissue. RXR-alpha also forms a heterodimer with Peroxisome Proliferator-Activated Receptor-gamma (PP AR-gamma), not shown on the KEGG pathway, which binds to promoter sites and directly stimulates transcription of adiponectin. Of note, this is the site of action of thiazolidinedione rosiglitazone, which activates the PP AR-gamma-RXR-alpha heterodimers bound to PPAR-gamma response elements in the adiponectin promoter [43]. PPAR-gamma was also found in our study to be downregulated in omental adipose (−1.632 89 fold change, P = 0.006 071 56), further increasing insulin resistance.

CD36 (FATCD36), found to be downregulated in omental adipose, is involved in the uptake of free fatty acids from the plasma [37,38]. ACSL1 (FAT) also downregulated in omental adipose in our study is responsible for activation of free fatty acids for utilization within the cell. Decreased activity of this enzyme decreases the rate of fatty acid storage. Notably, a study showed that the content/activity of fatty acid storage enzymes in omental fat was dramatically lower in those with more visceral fat [39]. Recent evidence supports the role of CD36 in storing fatty acids in adipose tissue. The actions of these proteins prevent the adverse physiologic effects from high circulating free fatty acid (FFA) levels [39,40]. High circulating FFA levels can also lead to pro-inflammatory processes that impair insulin signaling in liver, adipose, and skeletal muscle leading to insulin resistance [41,42]. It has been shown that CD36 membrane levels and turnover are abnormal in diabetes, resulting in dysfunctional fatty acid utilization. Also, variants in the CD36 gene have been shown to increase susceptibility to the metabolic syndrome increasing risk for diabetes and cardiovascular disease [40]. Thus, the downregulation of these two genes in omental adipose tissue may predispose to hyperlipidemia and its sequelae.

GLUT 1 was upregulated in omental adipose in our study. It functions as a basal constitutive glucose transporter, however GLUT-1 is minimally, if at all upregulated in the plasma membrane by insulin stimulation in skeletal muscle and adipocytes [44–46]. It has been widely observed that insulin-dependent glucose influx in skeletal muscle and adipocytes relies largely on GLUT-4, not GLUT-1. Moreover, it has been shown that insulin resistance in skeletal muscle and adipose is caused by abnormalities in glucose transport via GLUT-4 [47], which we did not find to be differentially expressed in our study. GLUT-1 was found to be downregulated in skeletal muscle of gestational diabetic women relative to that of normal glucose tolerant pregnant women with no differential expression in subcutaneous adipose tissue. Importantly, GLUT-4 was downregulated in both adipose and skeletal muscle of gestational diabetic women in the same study [48]. Of note, elevation of GLUT-1 expression in adipocytes has been observed in insulin resistant animal models. One study suggests this may be part of a compensatory mechanism for glucose entry in a condition in which normal insulin-stimulated glucose uptake is attenuated [49]. Another report noted that high fat feeding may link GLUT1 upregulation mechanistically via oxidative stress through chronic increased glucose influx to diet-induced insulin resistance [50]. Thus current evidence does not support a role for GLUT1 in insulin sensitivity in adipose tissue.

Carnitine palmitoyl transferase 1C (CPT1C) mRNA was found to be upregulated in omental adipose. The CPT1C isoform is atypical of CPT1 family proteins since it does not seem to be functionally related to CPT1A and CPT1B but seem to appear in the same category because it has high sequence similarity to them [51]. CPT1C has been shown to be primarily expressed in brain tissue; thus its upregulation noted in our study maybe a novel finding [52,53]. Unlike CPT1A and CPT1B, CPT1C is unable to catalyze acyl transfer from fatty acyl-CoAs to carnitine [53]. Recent studies have shown that CPT1c expression is localized in the ER and not in mitochondrial membrane which suggests that it may be less involved with fatty acid oxidation, and may be closer involved with palmitate transport across ER membrane, or perhaps as a metabolic sensor [51]. These findings underscore the poorly understood involvement of this gene in the regulation of energy homeostasis.

Limitations of this study include the utilization of a heterogeneous ethnic group. It is well accepted that risks of insulin resistance and diabetes may differ by ethnicity [54,55]. However our ANOVA analysis showed the minimal contribution of ethnicity to variation of the differently expressed genes. It is also possible that we could have used a larger sample size. However a review of the literature shows similar sample size in comparable studies [56–58]. We attempted to limit the influence of obesity on our study findings by excluding patients who are classified as obese with a BMI of 30; however we noted that some variation of our study may still be due to BMI since the F ratio was approximately 2 in the ANOVA analysis. A next step would be to perform metabolic testing to confirm function of the expressed genes.

It is acknowledged that adipose tissue is metabolically active and that intra-abdominal fat contributes to the pathophysiology of insulin resistance and inflammation [59]. Elucidation of the mechanisms of metabolic function of adipose tissue is ongoing. Most studies on adipose tissue are in the general population with fewer studies performed on pregnant women. The pathophysiology of adipose tissue function and insulin resistance in pregnancy is still not well understood, and may not necessarily be identical to that in the non-pregnant state. It is well accepted that obesity affects metabolic function, thus in this study we utilized only non-obese pregnant women in order to exclude the effect of obesity and assess the effect of pregnancy. In this study, we have shown the downregulation of several key genes in the KEGG Adipocytokine Pathway in omental when compared to subcutaneous adipose tissue, in which downregulation is linked to insulin resistance or inflammation. We observed borderline upregulation of GLUT-1, and have noted that the literature suggests this not have any significant role in insulin sensitivity in adipose tissue. As discussed, the upregulated gene CPT1C is poorly understood but we saw novel differential expression in omental adipose tissue. We believe that the findings from our study may contribute to literature on the metabolic activity of omental adipose tissue in pregnancy.

## Figures and Tables

**Table:** 

KEGG Gene Symbol	Official Gene Symbol(s)	Gene name(s)
	Downregulated genes	
FATCD36	CD36	CD36 molecule (thrombospondin receptor)
FACS	ACSL1, ACSL3, ACSL4, ACSL5, ACSL6	acyl-CoA synthetase long-chain family member 1, 3, 4, 5, and 6
IRS	IRS1, IRS2, IRS4	insulin receptor substrate 1, 2, and 4
SHP2	LOC344593 or LOC442113 or PTPN11	protein tyrosine phosphatase, non-receptor type 11
ADIPO	ADIPOQ	adiponectin, C1Q and collagen domain containing
RXR	RXRA, RXRB, RXRG	retinoid × receptor alpha, beta, and gamma
	Upregulated genes	
GLUT1/4	SLC2A1, SLC2A4	solute carrier family 2 (facilitated glucose transporter), member 1, solute carrier family 2 (facilitated glucose transporter), member 4
CPT-1	CPT1A, CPT1C, CPT1B or CHKB	carnitine palmitoyltransferase 1A (liver), carnitine palmitoyltransferase 1C, carnitine palmitoyltransferase 1B (muscle) or choline kinase beta

* Note where gene symbols/names are separated by a comma, this denotes different genes, but where they are separated by the word “or” this denotes they are synonyms.

** Downregulated/upregulated genes in each gene list are underlined.

**Table 1:** Primer sequences for PCR validation of microarray results.

GeneSymbol:	REFSEQ_ID:	5’ to 3’	Reverse primer:

		Forward primer:	
GAPDH	NM_002046	c a g g t g g t c t c c t c t g a c t t	c c a a a t t c g t t g t c a t a c c a
IL-6	NM_000600.3	g a a a g t g g c t a t g c a g t t t g a	g g t a a g c c t a c a c t t t c c a a g a
IL-1B	NM_000576	g g g c a a g a a g t a g c a g t g t c	g a g a g c a c a c c a g t c c a a a
CLDN1	NM_021101	c c t g g a t t t g a g t c t t g g t g	c c g c t t a c a g a t g a a g c a a t
ADIPOQ	NM_004797	t g g c t a t g c t c a c a g t c t c a	t c t c t c t g t g c c t c t g g t t c
CFB	NM_001710	c a g a c t a t c a g g c c c a t t t g	g t t g c t g g c a a g t g g t a g t t
IRS1	NM_005544	a c a c c g a g g a t a a a c a c t g g	c t a c g g a a g g a c c a c a a a g a

**Table 2:** Patient characteristics for study group.

	Race	Age	Gestational age	Birth weight	Maternal weight at delivery	BMI
Patient 1	Afr Am	33	40 + 0	3213	75.8	22.2
Patient 2	Afr Am	28	39 + 0	3950	77.1	25.5
Patient 3	Afr Am	41	39 + 1	3210	88.9	26.3
Patient 4	Asian	30	39 + 5	2760	59	20.7
Patient 5	Asian	31	38 + 2	3230	78.9	23
Patient 6	Caucasian	20	39 + 0	3400	75.4	20.8
Patient 7	Caucasian	38	39 + 0	3493	89	22.3
Patient 8	Caucasian	36	37 + 2	2850	78.4	24.9
Patient 9	Hispanic	37	39 + 0	2769	56.7	18.7
Patient 10	Hispanic	37	39 + 0	3420	58	21
Patient 11	Hispanic	26	39 + 1	3460	82.6	21.7
Patient 12	Hispanic	38	38 + 5	2630	77.1	23
Patient 13	Hispanic	32	39 + 0	3760	84.8	25.8
Patient 14	Hispanic	23	38 + 5	3210	95.3	29.7
Mean		32.1	39 + 0	3240	77	23.3
Standard deviation		6.21	4.37 days	384.7	12.3	2.88

Afr Am = African American; Age in years; Gestational Age in weeks + days; Birth weight in grams; Maternal weight at delivery in kilograms.

**Table 3:** Differentially expressed genes in omental relative to subcutaneous adipose tissue.

Gene symbol (Symbol in KEGG pathway)	Gene name	Fold-change	P-value	Function
IRS1 (IRS)	Insulin Receptor Substrate-1	−1.771 63	0.000 014 6	Interacts with insulin receptor and enables insulin metabolic actions by signaling PI3K pathway
ADIPOQ (ADIPO)	Adiponectin, C1Q and collagen domain containing	−1.667 34	0.039 814	Signals increased glucose uptake via the GLUT transporters, modulates fatty acid catabolism
RXRA (RXR)	Retinoid × Receptor, alpha	−1.614 26	0.000 015	Activation of PPAR-alpha to promote uptake and utilization of fatty acids
CD36	Thrombospondin receptor	−2.058 39	0.000 181	Uptake of free fatty acids (FFA) from plasma
ACSL1 (FACS)	Acyl-CoA synthetase long-chain family member 1	−1.701 68	0.003 205	FFA activation for triacylglycerol synthesis
PTPN11 (SHP2)	Protein tyrosine phosphatase, non-receptor type 11	−1.733 8	0.0 000 256	Has regulatory role in mitogenic activation, metabolic control, transcription regulation, and cell migration
SLC20A1 (GLUT1)	Solute carrier family 2(facilitated glucose transporter), member 1	1.589 51	0.000 079 4	Plasma membrane glucose transporter, responsible for basal glucose intake In adipose and skeletal muscle
CPT1C (CPT-1)	Carnitine palmitoyltransfer-ase 1C	1.703 3	0.000 060 2	Function not well understood, believed to play a role in regulation of energy homeostasis

**Table 4:** Real-time PCR validation of microarray results.

		Fold Change	
Gene	Ref_seq	Microarray	RT-PCR
IL-6	NM_000600	2.19	1.23
IL-1B	NM_000576	2	1.31
CLDN1	NM_021101	18.61	13.89
ADIPOQ	NM_004797	−1.67	−1.58
CFB	NM_001710	10.62	11.6
IRS1	NM_005544	−1.77	−1.48
PLA2G2A	NM_000300	4.48	3.5
